# Exploring ChatGPT’s Potential in Facilitating Adaptation of Clinical Guidelines: A Case Study of Diabetic Ketoacidosis Guidelines

**DOI:** 10.7759/cureus.38784

**Published:** 2023-05-09

**Authors:** Ehab Hamed, Ahmad Eid, Medhat Alberry

**Affiliations:** 1 Qatar University Health Center, Primary Health Care Corporation, Doha, QAT; 2 Umm Slal Health Center, Primary Health Care Corporation, Doha, QAT; 3 Fetal Medicine, Sidra Medicine, Doha, QAT

**Keywords:** ai chatbot, healthcare technology, evidence-based medicine, evidence-based recommendations, chatgpt, medical informatics, healthcare management, prompt design, artificial intelligence, clinical guidelines

## Abstract

Background

This study aimed to evaluate the efficacy of ChatGPT, an advanced natural language processing model, in adapting and synthesizing clinical guidelines for diabetic ketoacidosis (DKA) by comparing and contrasting different guideline sources.

Methodology

We employed a comprehensive comparison approach and examined three reputable guideline sources: Diabetes Canada Clinical Practice Guidelines Expert Committee (2018), Emergency Management of Hyperglycaemia in Primary Care, and Joint British Diabetes Societies (JBDS) 02 The Management of Diabetic Ketoacidosis in Adults. Data extraction focused on diagnostic criteria, risk factors, signs and symptoms, investigations, and treatment recommendations. We compared the synthesized guidelines generated by ChatGPT and identified any misreporting or non-reporting errors.

Results

ChatGPT was capable of generating a comprehensive table comparing the guidelines. However, multiple recurrent errors, including misreporting and non-reporting errors, were identified, rendering the results unreliable. Additionally, inconsistencies were observed in the repeated reporting of data. The study highlights the limitations of using ChatGPT for the adaptation of clinical guidelines without expert human intervention.

Conclusions

Although ChatGPT demonstrates the potential for the synthesis of clinical guidelines, the presence of multiple recurrent errors and inconsistencies underscores the need for expert human intervention and validation. Future research should focus on improving the accuracy and reliability of ChatGPT, as well as exploring its potential applications in other areas of clinical practice and guideline development.

## Introduction

Artificial intelligence (AI) has become increasingly important in healthcare due to its potential to improve patient care and outcomes. From diagnosis to treatment and management of various health conditions, AI has shown promise in a wide range of applications [1]. Large language models (LLMs) and natural language processing (NLP) are of particular interest to the medical field as they have the potential to assist in the adaptation of clinical guidelines. Clinical guidelines provide evidence-based recommendations to guide the diagnosis, treatment, and management of different health conditions, but their development is a resource-intensive process. Adapting these guidelines to reflect the latest scientific evidence and local contexts may be less resource-intensive but can be a complex process.

ChatGPT, an AI chatbot that uses NLP, can extract, summarize, compare, and contrast information from different guidelines and integrate findings into a comprehensive guideline [2]. Language models such as ChatGPT have demonstrated the potential to assist in medical academic research and clinical decision-making throughout the clinical workflow, from triage to diagnosis to management [3,4]. However, it is important to note that ChatGPT may generate incomplete, inconsistent, or irrelevant information that does not match user intentions or expectations [5,6].

Prompt engineering is a tool used to optimize the accuracy and precision of the output from LLMs and NLP models. It involves designing and implementing prompts or task-specific instructions to guide the model’s responses. By providing prompts that specify the desired output, prompt engineering can reduce the likelihood of the model generating irrelevant or incorrect responses. In addition, it allows for greater control over the model’s output as the prompts can be tailored to suit the specific task at hand. Studies have shown that prompt engineering can significantly improve the performance of LLMs and NLP models. The use of prompt design techniques has been shown to improve the quality and accuracy of ChatGPT’s output in various applications [7-9]. Therefore, prompt engineering techniques are implemented to maximize the reliability and consistency of ChatGPT’s output [9-12]. However, it is important to recognize that prompt design alone is not enough to ensure consistency and reliability of the output [13,14]. Prompt components and structure are vital to ChatGPT’s output [15].

In our case study, we focused on diabetic ketoacidosis (DKA) as a prime example. Multiple clinical guidelines for treating DKA provide a comprehensive coverage of the topic and an array of options for healthcare professionals. However, these guidelines may have been developed for specific contexts or populations, which can limit their direct applicability in other settings or groups [16-18].

National clinical guidelines represent the gold standard in medical practice in each nation, but their development is time-consuming and resource-intensive. They are typically developed by national healthcare organizations or government agencies. Creating new guidelines for low- and middle-income (LMIC) settings can be costly and challenging. An alternative approach is to leverage existing clinical practice guidelines (CPGs) as a basis for adapting and formulating locally relevant recommendations. CPGs offer evidence-based and authoritative guidance for optimal patient care [19], and AI tools such as ChatGPT may facilitate their adaptation to suit diverse healthcare settings. While AI tools require pre-programming to ensure accuracy and reliability, the use of prompt engineering techniques may help to tailor their output to suit specific healthcare settings and practitioner needs.

The ADAPTE framework serves as a methodological instrument, enabling guideline developers and users to systematically select, evaluate, and tailor existing CPGs for a specific setting. Comprising three primary phases, namely, set-up, adaptation, and finalization, the ADAPTE framework offers a structured approach to the adaptation of clinical guidelines.

The adaptation phase encompasses extracting recommendations from chosen CPGs, evaluating their relevance and applicability to the target context or population, modifying or generating new recommendations as necessary, and grading the strength of these recommendations. The finalization phase involves validating the adapted CPGs with external experts and stakeholders, formatting and disseminating the adapted CPGs, and strategizing their implementation and evaluation [20]. The dependability of the ADAPTE framework for adapting guidelines hinges on the credibility of the sources employed.

Clinical guidelines developed through systematic reviews are particularly advantageous for the adaptation process as they provide a comprehensive and reliable source of recommendations based on the latest scientific evidence. The systematic review process ensures that all relevant studies are identified, critically appraised, and synthesized to provide an evidence base for the guideline. Guidelines developed through systematic reviews are widely used in resourceful healthcare systems and represent the gold standard for the development of clinical guidelines. AI tools such as ChatGPT can facilitate the adaptation and tailoring of existing guidelines to meet the specific needs of local healthcare settings and populations. Regarding the location of use for AI tools such as ChatGPT, the technology can be used in any geographic location with access to the necessary infrastructure and resources.

The efficacy of the ADAPTE framework can be bolstered by integrating NLP and LLM techniques to enhance the efficiency and accuracy of guideline adaptation. These tools can autonomously extract and compare recommendations from multiple sources, resulting in comprehensive and inclusive guidelines that consider both evidence and context. Additionally, NLP and LLM techniques can streamline the adaptation process by creating summaries, visualizations, and interactive dialogues that engage diverse stakeholders. Nevertheless, it is essential to conduct empirical studies to evaluate the feasibility, validity, and impact of such an approach.

In addressing this gap, our study aims to investigate the use of NLP and LLM techniques in conjunction with the ADAPTE framework comparison component to synthesize guidelines for managing DKA. We hypothesize that, by employing clear prompting and specifying reliable sources, AI tools can facilitate guideline adaptation by generating consistent and reliable guidelines that incorporate recommendations from multiple sources while reducing human error and effort. By showcasing ChatGPT’s potential as a language model for synthesizing medical information, our study contributes to the field of medical informatics and provides practical guidelines for managing DKA that can be implemented by healthcare providers in various settings. Acknowledging the limitations of NLP and LLM, we emphasize the continued necessity for human validation of the generated output.

## Materials and methods

Study design

We used a systematic and structured approach to adapt clinical guidelines for the management of DKA using the ChatGPT language model by Open AI. The process involved designing and trialing ChatGPT prompts for the task until they were optimized, identifying current national clinical guidelines, defining simple clinical questions, extracting and comparing answers using ChatGPT, integrating information into a unified guideline, and reviewing the output against the original text.

To ensure consistent and reliable content, we designed a ChatGPT prompt for each clinical question and conducted multiple experiments using the ChatGPT 4.0 version. Throughout the process, we experimented with different prompts to identify the most effective approach for our specific use case. The results of our experiments were evaluated for accuracy and output format, and we conducted additional experiments to explore consistency. We acknowledge that the current stage of ChatGPT development presents limitations for achieving completely reliable and consistent results, and our study aimed to test the hypothesis that prompt engineering could help to improve the accuracy and consistency of ChatGPT’s output.

Incorporating three guidelines helped mitigate potential misinterpretations or misreporting by the language model. Criteria for selection included guidelines that were current, nationally used, and developed through systematic reviews. The three evidence-based clinical guidelines chosen for adaptation were the Diabetes Canada Clinical Practice Guidelines Expert Committee, The Royal Australian College of General Practitioners and Australian Diabetes Society position statement, and The Joint British Diabetes Societies (JBDS) guidelines (Table 1).

**Table 1:** Steps of applications with useful directives.

Steps	Useful directives
Use prompts to search for and identify relevant clinical guidelines (ChatGPT and human supervision)	Select guidelines from reliable sources (e.g., professional associations, government agencies, etc.). Ensure guidelines are up-to-date and evidence-based. Human supervision to confirm the reliability and relevance of the selected guidelines. Save the list of the references for usage in all the following clinical question prompts
Define clinical questions (Human)	Identify key aspects of the clinical condition or topic (e.g., diagnosis, risk factors, signs/symptoms, investigations, treatment). Formulate clear and concise questions to address each aspect
Use prompts for retrieval and comparison with one clinical question at a time (ChatGPT and human supervision)	Extract the relevant information from the AI-generated responses. Compare and contrast the information with different guidelines. Verify accuracy (human)
Integrate information into a unified guideline, summarize, and review (ChatGPT and human supervision)	Combine the extracted information to create a comprehensive guideline. Review areas of disagreement between guidelines. Review and verify accuracy (human). Present to stakeholders for review and adaptation

In designing prompts, we considered several factors. We crafted clear and specific instructions, employed appropriate punctuation, and broke down the process into distinct steps while establishing the role of AI as an expert in guideline adaptation. The action required by the prompt, such as comparing, was explicitly stated. We also unambiguously indicated the preferred output format, such as a table or summary. To ensure the AI focused on the selected guidelines, constraints were applied to limit references to a specific set. If the generated response did not provide accurate or relevant information, the prompts were revised for clarity and repeated until a suitable response was generated. The suitability of the response was ultimately determined by the authors who assessed whether the response provided accurate and relevant information to answer the clinical question at hand.

By adhering to these factors, we produced the final three prompts to standardize the extraction process for this study (Table 2).

**Table 2:** ChatGPT tasks and prompts.

Task	Prompt
Search and list guidelines	As an expert AI search engine for national or international clinical guidelines, your task is to provide a comprehensive list of all national and international clinical guidelines for the topic of [condition] that have been published or updated after [date]. You use your expertise and extreme intelligence to achieve your task. You cannot say I am not able to, or I do not have the capability. To complete this task, follow these guidelines: 1. Use reputable sources such as PubMed, Cochrane Library, or guideline databases to search for clinical guidelines on [condition] that have been published or updated after [date]. List of Clinical websites to search. a. https://pubmed.ncbi.nlm.nih.gov/ b. https://www.nice.org.uk/guidance c. https://www.sign.ac.uk/ d. https://www.nccih.nih.gov/health/providers/clinicalpractice e. https://www.who.int/publications/who-guidelines f. https://www.uspreventiveservicestaskforce.org/uspstf/ g. https://guidelines.ebmportal.com/ h. https://joulecma.ca/cpg/homepage. 2. Check that each guideline is national or international and relevant to the topic of [condition]. 3. Use the Vancouver citation style to format the list of guidelines. Make sure to include the author(s), title of the guideline, name of the organization that published the guideline, year of publication, and date accessed if applicable. 4. Organize the list of guidelines in chronological order, with the most recent guidelines listed first. By following these guidelines, you can provide a comprehensive and up-to-date list of national and international clinical guidelines
Retrieve and define clinical guidelines	As an expert in reporting on clinical guidelines content, your task is to create a comprehensive table that reports on medical clinical guidelines for Diabetic Ketoacidosis (DKA) by following these steps: Review the three DKA clinical guidelines from the provided list of references [References A-C]. Anticipate a specific clinical question related to DKA. Extract pertinent information from each guideline to address the clinical question, ensuring accuracy and noting any special cases mentioned. Develop a table summarizing the guideline recommendations with columns: Points to Cover, Guideline 1, Guideline 2, Guideline 3, and Cumulative Guidelines (combining data from all three columns). Use the guidelines’ names as headings. In the last column, include all data from the three columns as they are but ensure no repetition. Follow these constraints: a. Only utilize the three clinical guidelines provided. b. Ensure the table addresses all relevant points related to the DKA clinical question. c. Review the table against the guidelines to enhance accuracy and confidence in the summary prior to final production. Please confirm your understanding of these instructions and your readiness to proceed with the task. References: A. Diabetes Canada Clinical Practice Guidelines. URL: https://guidelines.diabetes.ca/cpg/chapter15 B. Emergency Management of Hyperglycaemia in Primary Care - RACGP and ADS Joint Clinical Position Statement. URL: https://www.racgp.org.au/getattachment/ebb0683e-fed4-4b90-b0bb-e4f353399386/Management-of-hyperglycaemia.pdf.aspx C. The Management of Diabetic Ketoacidosis in Adults—An Updated Guideline from the Joint British Diabetes Society for Inpatient Care. Diabetic Medicine. URL: https://onlinelibrary.wiley.com/doi/epdf/10.1111/dme.14788
Answering clinical question	Clinical question, e.g., What are the diagnostic criteria for [condition] in each of the guidelines?

Ethical considerations

The study focused on the adaptation of publicly available clinical guidelines and did not involve human subjects or patient data. No ethical approval was required for this study.

Bias and confounding

The authors designed the algorithm and determined reliable sources for the guidelines. The guidelines were chosen based on two main criteria, namely, development through a systematic review process and currency. The authors prioritized guidelines from reliable sources such as national or international medical organizations, professional societies, and government agencies. These guidelines are widely recognized as authoritative and are typically based on the most up-to-date, evidence-based recommendations.

While the authors did consider additional guidelines beyond the three used in the study, only those that met the inclusion criteria were included in the analysis. The authors recognize that potential confounding factors, such as the specific clinical context or the expertise of the healthcare providers, were beyond the scope of the study.

Justification of study design

The systematic and structured approach employed in this study facilitated standardized, efficient, and accurate reporting, capitalizing on the capabilities of the ChatGPT language model while ensuring human supervision in selecting guidelines, formulating clinical questions, and validating the output. We employed a rigorous review process to assess the accuracy and precision of ChatGPT’s output, including individual components and the final synthesized guidance.

In conclusion, the methods employed in this study provided a systematic and structured approach to assess the process of adapting clinical guidelines using the ChatGPT language model. The algorithm and its associated steps enabled the efficient extraction, comparison, and integration of information from multiple guidelines, allowing for a comprehensive evaluation.

## Results

This study assessed ChatGPT’s effectiveness in adapting clinical guidelines for DKA management by comparing and consolidating information from three diverse sources. ChatGPT produced a remarkable and comprehensive table of the questions asked, covering most of the relevant information. This may be because the prompt instructions were clear and specific, guiding the model to focus on the key aspects of the questions. It may also be because the model used the gold standard of medical knowledge, which included three current national guidelines that provided consistent and authoritative information.

However, the table also contained some errors, such as reporting diagnostic criteria incorrectly in some guidelines and omitting a risk factor. These errors made the table unreliable and potentially misleading. Additionally, the table content varied inconsistently on the regeneration of output, leading to further inconsistencies. Therefore, factors contributing to the observed inaccuracies may include misinterpretation of source material, incomplete information extraction, ambiguity in the source material, and training data limitations.

The limitations observed in ChatGPT’s capacity to accurately process and report complex medical information reveal potential challenges in using AI-driven models such as ChatGPT in adapting clinical guidelines for medical practice. Despite these limitations, ChatGPT demonstrated potential in consolidating clinical guidelines, offering a basis for further development and improvement. Previous studies have shown that prompt structure and wording, attention mechanisms, and data quality can affect the performance of LLMs and NLP techniques applied to the task of extracting and summarizing clinical guidelines [8,9]. Future research should explore these factors and methods to enhance the accuracy and reliability of AI-generated content in medicine, especially when dealing with complex and critical information (Table 3).

**Table 3:** Observations and limitations of language model performance in extracting information from diabetic ketoacidosis (DKA) clinical guidelines.

Category	Observation/Error	Description
Performance observations	Prompt dependency	The language model’s output is significantly influenced by the structure, wording, and punctuation of prompts. Clear and precise prompts are necessary for obtaining accurate and relevant information
	Enhanced data quality	Providing direct links to guidelines, detailed instructions, and varied terminology (e.g., “risk factors” and “precipitating factors”) results in more accurate and detailed responses
	Value of focused versus general questions	Focused questions lead to more detailed answers (e.g., fluid treatment advised for DKA vs. general treatment for DKA)
	Handling ambiguity	ChatGPT struggles with handling unclear or conflicting information from the source material, such as diagnostic criteria
Errors and limitations	Incomplete information extraction	ChatGPT does not always include all relevant information from the source material, such as ignoring euglycemic DKA in all outputs
	Unwarranted information addition	ChatGPT added a list of medications to those that could cause DKA, which were not present in the original guidelines
	Unreliable quoting or referencing	When asked to quote the source of information, the model quoted text that was not present in the original guidelines
	Inconsistent outputs	ChatGPT generated different answers for the same question on different runs, occurring mainly with ambiguous prompts

Implications

The study findings have significant implications for the use of AI-generated content in medical practice as incorrect diagnoses and inappropriate treatment decisions may harm patient health. To address ChatGPT’s limitations, practical steps can be taken, such as enhancing the quality and quantity of training data, refining the algorithms for medical information analysis, and incorporating visual aids such as tables and figures to improve understanding and identify areas for improvement. These steps would help increase the accuracy and reliability of AI-generated content, thus promoting accessibility and dissemination of clinical guidelines in the medical field.

Limitations

The study reported on a process with one clinical topic and three clinical guidelines. Although ChatGPT demonstrated potential in consolidating clinical guidelines, the unexpected errors in the generated table raise concerns about its reliability for guideline adaptation. While our study demonstrates the potential of ChatGPT for medical guideline adaptation, further research with larger sample sizes and a more rigorous methodology is necessary to fully investigate its capabilities and limitations. Collaboration between all users, including medical professionals and software developers, is crucial for the successful implementation of AI tools such as ChatGPT in the medical field. Ongoing research and collaboration will help to improve the reliability and consistency of ChatGPT’s output and ensure its effectiveness in facilitating medical guideline adaptation.

ChatGPT unexpected or contradictory results

Unexpected errors in the generated table, such as misreporting diagnostic criteria or omitting the odd risk factor, raise concerns about ChatGPT’s reliability for guideline adaptation. Possible explanations for these inconsistencies include limitations in the model’s training data and the complexity of the source material. The study results emphasize the need for careful interpretation and verification of information generated by ChatGPT. Further studies with larger sample sizes and more rigorous methodology are required to investigate ChatGPT’s capabilities and limitations in medical guideline adaptation. Future research should also explore methods to enhance the accuracy and reliability of AI-generated content in the medical field, especially when handling complex and critical information.

## Discussion

The study’s findings demonstrate the potential application of AI tools, such as the ChatGPT language model, in facilitating the adaptation of clinical guidelines. By extracting, comparing, and integrating recommendations from reliable sources, the study developed a comprehensive and up-to-date guideline for the management of DKA [16-18]. While the use of ChatGPT produced a remarkable and comprehensive table of the questions asked, the table also contained errors, which made it unreliable and potentially misleading. Factors contributing to the observed inaccuracies may include misinterpretation of source material, incomplete information extraction, ambiguity in the source material, and training data limitations. These limitations reveal potential challenges in using AI-driven models such as ChatGPT in adapting clinical guidelines for medical practice.

In our study, we used a methodology based on the ADAPTE framework to systematically select, evaluate, and tailor existing clinical practice guidelines for the adaptation process. We designed ChatGPT prompts to ensure consistent and reliable content and evaluated the results for accuracy and output format. We also conducted additional experiments to explore consistency. The methodology employed in the study could be applied to other medical topics, enhancing the efficiency of guideline development and dissemination in the medical field.

However, alternative explanations for the results could include the influence of the quality and clarity of the input guidelines and clinical questions provided, as well as the limitations of the ChatGPT model in understanding complex medical jargon or addressing nuances in clinical practice. The study’s limitation includes the reliance on human supervision for the selection of appropriate guidelines, the formulation of clinical questions, and the validation of results. The study focused solely on the guidelines provided and may not cover all aspects of DKA management, and the clinical context and expertise of healthcare providers were not considered, which may affect the applicability of the adapted guideline in real-world settings.

The study’s findings have implications for future research, clinical practice, and policymaking. Future research could explore the application of the ChatGPT model or other AI tools in the adaptation of clinical guidelines across a broader range of medical topics. In clinical practice, AI-assisted guideline adaptation could lead to more efficient dissemination of up-to-date, evidence-based recommendations, potentially improving patient care and outcomes. For policymaking, incorporating AI tools in the development and dissemination of clinical guidelines may contribute to more informed and effective decision-making in healthcare.

Overall, the study demonstrates the potential of AI tools, such as the ChatGPT language model, in the adaptation of clinical guidelines. While the study has limitations, the findings contribute valuable insights into the potential applications of AI in the medical field, particularly in the development and dissemination of evidence-based clinical guidelines. The study provides a proof of concept for further research and reports. Further research is needed to explore the broader implications of AI-assisted guideline adaptation for various medical topics and settings. The use of AI tools such as ChatGPT has the potential to enhance the process of clinical guideline adaptation, addressing gaps and challenges in the field. The study adds to the growing body of evidence supporting the integration of AI in medical practice and provides a foundation for future research aimed at refining and expanding the application of AI tools for guideline adaptation purposes.

## Conclusions

This study evaluated ChatGPT’s effectiveness in adapting clinical guidelines for DKA management by comparing and consolidating information from three diverse sources. The results showed that ChatGPT was able to produce a remarkable and comprehensive table of the questions asked, covering most of the relevant information. However, the results also revealed some errors and limitations in ChatGPT’s output, such as misreporting diagnostic criteria, omitting a risk factor, varying content on regeneration, misinterpreting source material, incomplete information extraction, ambiguity in the source material, and training data limitations. These errors and limitations cast doubt on ChatGPT’s trustworthiness in adapting clinical guidelines for medical practice and highlight the need for cautious interpretation and validation of AI-generated content in medicine. The study also proposed some explanations for ChatGPT’s performance and limitations, such as prompt structure and wording, attention mechanisms, and data quality. The study demonstrated ChatGPT’s potential in consolidating clinical guidelines, offering a basis for further development and improvement. Future research should explore these factors and methods to enhance the accuracy and reliability of AI-generated content in medicine, especially when dealing with complex and critical information.
